# Reversible Cerebral Vasoconstriction Disorder in a Patient with a Chief Complaint of Headache

**DOI:** 10.7759/cureus.2487

**Published:** 2018-04-16

**Authors:** Simran Buttar, Anuja Trivedi, Dimitrios Papanagnou

**Affiliations:** 1 Emergency Medicine, Maimonides Medical Center; 2 Capital Health, Capital Health Medical Center; 3 Department of Emergency Medicine, Thomas Jefferson University

**Keywords:** reversible cerebral vasoconstriction disorder, neurological symptoms, headache

## Abstract

As emergency medicine physicians, we have formulated an approach to managing patients with a chief complaint of headache that starts with considering the story the patient relays in the context of a wide differential. Here we will describe a case that presented to our emergency department in hopes to broaden your differential. Reversible cerebral vasoconstriction syndrome (RCVS), well described in the neurology literature, is characterized by severe headaches that may or may not be accompanied by neurological symptoms and is definitively diagnosed by diffuse constriction of cerebral arteries on cerebral angiogram. Here we present a case of a patient who presented to the emergency department with intermittent severe persistant headaches and was diagnosed with reversible cerebral vasoconstriction syndrome.

## Introduction

Reversible cerebral vasoconstriction syndrome (RCVS) is a recognized syndrome within the neurology and cerebrovascular community. Our goal with this case report is to bring it to the attention of the emergency medicine community as well. The clinical presentation of this syndrome is “sudden onset and severe headaches over one to three weeks, often accompanied by nausea, vomiting, photophobia, confusion, and blurred vision” [1]. Definitive diagnosis requires a cerebral angiogram showing the characteristic “string of beads” [1], which demonstrates the diffuse vasoconstriction of the cerebral arteries. The treatments proposed by various sources have not shown to prevent the more significant ischemic or hemorrhagic complications, although there is a lack of randomized control trials in this area [1]. The case we present here was one that we found perplexing and resulted in this diagnosis, which we had no prior education on.

## Case presentation

A 49-year-old woman with a past medical history of hypertension, preeclampsia, anxiety, and bipolar disorder on buspirone, presented to the emergency department with triage complaint of “multiple complaints”. Her history of present illness revealed a persistent headache that initially started one week prior to arrival. The patient recalled onset in the evening associated with nausea and vomiting. She went to an urgent care the next day and received medications, after which she felt better for about two days. When her symptoms recurred, she went to an outside hospital where she had a computed tomography scan of the head and lumbar puncture, both of which were negative. The patient was admitted for an elevated troponin level and received a cardiac catheterization without intervention, findings significant for 60% blockage of a single vessel. The patient presented to us one day post discharge due to persistent headache. She described the headache as similar to her prior preeclampsia headache, feeling "like a grip around" her entire head.

Her initial vital signs were as follows: blood pressure 172/92 mmHg, pulse 81, respiratory rate 18, and oxygen saturation 99% on room air. A physical exam revealed a woman in pain but nontoxic appearing. Her heart and lung sounds were normal. Her neurological exam was unremarkable with no focal numbness, weakness, or abnormalities with coordination, gait, or cranial nerves. The chest plain film, initial lab, and electrocardiogram results were normal.

The patient received one liter intravenous fluids, metoclopramide intravenous, and ketorolac intravenous with minimal improvement and still appeared uncomfortable on multiple reassessments. The patient was placed in the observation unit and a magnetic resonance imaging (MRI) of the brain was ordered.

**Figure 1:**
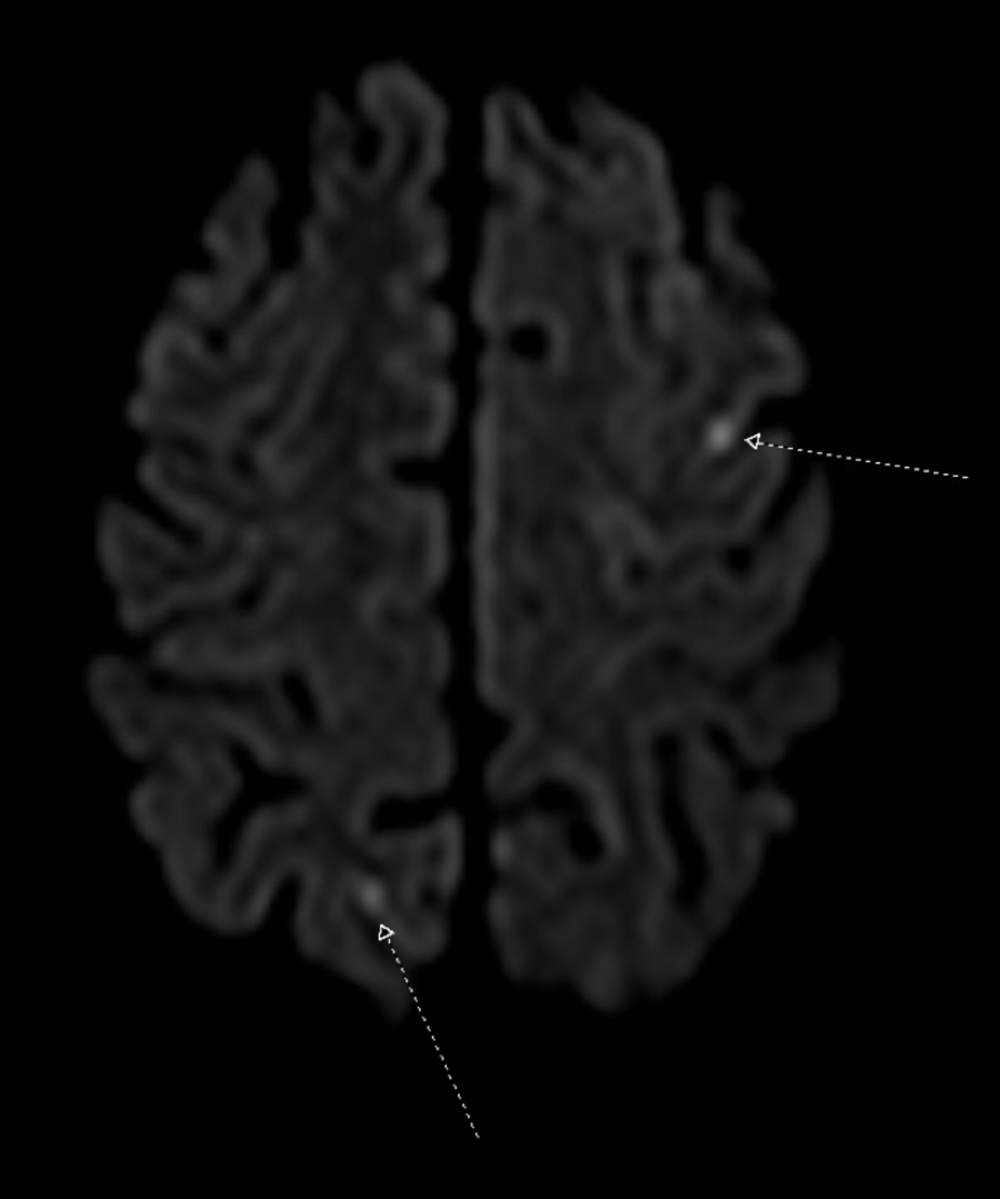
Magnetic Resonance Imaging Brain Noncontrast This image demonstrates two regions of focal restricted diffusion in the right parietal lobe and the left temporal lobe, which represent acute embolic infarcts.

The magnetic resonance imaging (MRI) of the brain showed three punctate regions of focal restricted diffusion in the left middle frontal gyrus, right parietal lobe, and left temporal lobe that appeared consistent with acute infarcts. The image is provided below (Figure 1).

Neurology was consulted and the patient was admitted to the stroke service. Her stroke workup, which included computed tomography angiography of the head and neck, transthoracic echocardiogram, and a transesophageal echocardiogram, was unremarkable. The differential at that point was narrowed down to vasculitis versus RCVS as the cause of the severe headache associated with acute infarction. Neurosurgery was consulted and an angiogram was performed, showing evidence of RCVS in the middle cerebral artery, M1 segment, and distal middle cerebral artery territories as well as the right posterior cerebral artery and distal anterior cerebral artery. Dual antiplatelet therapy with aspirin and clopidogrel was initiated and the patient was discharged to follow up with the stroke clinic and her primary care provider.

## Discussion

Reversible cerebral vasoconstriction disorder is becoming increasingly prominent as an interesting and more common than previously suggested diagnosis in the neurology literature. The etiology of this syndrome is thought to be due to a “disturbance in the control of cerebrovascular tone” [2]. It is difficult to make this diagnosis because of the transitory nature of findings on radiology studies. Initial negative brain imaging studies followed by an evidence of brain ischemia or hemorrhage found on subsequent imaging a few days later, as observed in this case, is the classic presentation of this syndrome [2]. The main diagnoses to exclude when considering RCVS in the differential are subarachnoid hemorrhage and vasculitis [3].

The majority of the cases of RCVS are patients who are either postpartum or on vasoactive drugs. The three patients Singhal et al. described who developed “thunderclap headache, reversible cerebral arterial vasoconstriction, and ischemic stroke” were all exposed to serotonin-enhancing drugs, which are thought to cause “reversible, multifocal arterial narrowing” [4]. In their analysis of 139 cases of RCVS, Singhal et al. found that 42% were exposed to vasoactive drugs such as cannabis, cocaine, amphetamines, selective serotonin reuptake inhibitors, and alpha sympathomimetics [5]. Our patient, in this case, was on buspirone, which is an agonist at serotonergic receptors. In RCVS, intracranial hemorrhage is frequent [6]. Women and patients with a history of migraines appear to be more at risk for intracranial hemorrhage in those with RCVS [6].

Little is known about the treatment of this condition. Both Ducros et al. and Singhal et al. have considered nimodipine in the treatment of the headaches associated with RCVS. There is currently no randomized control trial that has explored this treatment option and further research must be done to find a suitable therapy [2].

## Conclusions

As emergency medicine physicians, it is our responsibility to continually expand our differential for common chief complaints. Whether or not to include reversible cerebral vasoconstriction disorder in our differential for the patient with headache is something that warrants discussion. RCVS should be suspected in patients with recurrent thunderclap headaches, particularly in the setting of vasoactive drug use or postpartum state. Including RCVS in the differential for patients with recurrent headaches with vague neurological complaints is important in establishing their diagnosis and managing their symptoms. While the pathological process, definitive diagnosis, and treatment remain unclear, considering the diagnosis is an important first step in the care of these patients.
